# Quantifying Pharmaceutical Film Coating with Optical Coherence Tomography and Terahertz Pulsed Imaging: An Evaluation

**DOI:** 10.1002/jps.24535

**Published:** 2015-06-17

**Authors:** Hungyen Lin, Yue Dong, Yaochun Shen, J Axel Zeitler

**Affiliations:** 1 Department of Chemical Engineering and Biotechnology, University of CambridgeCambridge, CB2 3RA, UK; 2 Department of Electrical Engineering and Electronics, University of LiverpoolLiverpool, L69 3GJ, UK

**Keywords:** coating, imaging methods, physical characterization, process analytical technology (PAT), tablet, Processing

## Abstract

Spectral domain optical coherence tomography (OCT) has recently attracted a lot of interest in the pharmaceutical industry as a fast and non-destructive modality for quantification of thin film coatings that cannot easily be resolved with other techniques. Because of the relative infancy of this technique, much of the research to date has focused on developing the in-line measurement technique for assessing film coating thickness. To better assess OCT for pharmaceutical coating quantification, this paper evaluates tablets with a range of film coating thickness measured using OCT and terahertz pulsed imaging (TPI) in an off-line setting. In order to facilitate automated coating quantification for film coating thickness in the range of 30–200 μm, an algorithm that uses wavelet denoising and a tailored peak finding method is proposed to analyse each of the acquired A-scan. Results obtained from running the algorithm reveal an increasing disparity between the TPI and OCT measured intra-tablet variability when film coating thickness exceeds 100 μm. The finding further confirms that OCT is a suitable modality for characterising pharmaceutical dosage forms with thin film coatings, whereas TPI is well suited for thick coatings. © 2015 The Authors. *Journal of Pharmaceutical Sciences* published by Wiley Periodicals, Inc. and the American Pharmacists Association J Pharm Sci 104:3377–3385, 2015

## INTRODUCTION

The process of coating one or more layers of polymer onto tablets is almost ubiquitous in pharmaceutical manufacturing in order to achieve uniformity of colour, light protection, taste masking and, more recently, in advanced coatings such as active and sustained release, where the drug release kinetics can be controlled, thereby increasing the therapeutic efficacy of tablets.^1^ Pharmaceutical film coating is typically performed in large batches and the quality of the resulting product is largely affected by the uniformity of the tablet mixing dynamics that in turn is driven by tablet properties (e.g., size and shape), process parameters (e.g., pan speed, pan loading) and device-specific parameters (e.g., size, geometry and baffles, etc.). In an effort to better understand the complex interplay between the parameters while ensuring a consistent coating quality in advanced dosage forms, various techniques have been devised and demonstrated for characterising pharmaceutical film coating. A standard technique is obtaining the averaged weight gain of randomly selected tablets from a tablet batch. However, as weight gain is an aggregate metric, it cannot provide information specific to each dosage form such as film coating thickness and uniformity.^2^ To date, several non-destructive techniques have been explored to overcome this limitation. These include spectroscopic methods such as near-infrared and Raman spectroscopy^3^,^4^ and imaging methods such as nuclear magnetic resonance imaging,^5^ terahertz pulsed imaging (TPI)^6^,^7^ and X-ray micro-tomography.^8^,^9^ More recently, spectral domain optical coherence tomography (SD-OCT) has been demonstrated for at-line/off-line^10^–^12^ and in-line applications^13^ for pellet dosage forms as well.^14^,^15^ With the growing popularity of SD-OCT for pharmaceutical film coating quantification, especially with the recent proposal of an image processing-based automated coating quantification algorithm for in-line settings,^16^ this paper evaluates tablets with a range of film coating thickness as measured using SD-OCT assessed by TPI in an off-line setting in order to better understand the thickness limit that is quantifiable with SD-OCT. The present article aims to bring awareness to the pharmaceutical community, who may be interested in using SD-OCT for quantifying pharmaceutical film coatings.

## MATERIALS AND METHODS

### Tablet Production

The samples used in the present work comprise of a batch of pharmaceutical tablets with a single sustained-release polymer coating layer whereby the tablet cores were biconvex shaped and contained 10% (w/w) diprophyllin (API), 84.5% (w/w) lactose monohydrate (Flowlac® 100), 5% (w/w) vinylpyrrolidone–vinyl acetate copolymer (Kollidon®V64) and 0.5% (w/w) magnesium stearate. The transparent coating suspension has the following formulation: 50% (w/w) polyvinyl acetate (Kollicoat® SR 30D), 6% (w/w) polyvinyl alcohol–polyethyleneglycol graft copolymer (Kolicoat® IR), 0.075% (w/w) polyoxyethylene(20) sorbitan monooleate (polysorbate 80), 0.3% (w/w) glycerolmonostearate, 0.75% (w/w) triethylcitrate and 42.87% (w/w) deionised water. The tablet cores were coated in a pilot-scale coater BFC25, Bohle Film Coater (L.B. Bohle, Ennigerloh, Germany). The coating pan dimensions were 546 mm in diameter and 630 mm in length and the batch size was 20 kg. The coater used five two-way spray nozzles (970/7-1 S75; Düsen-Schlick GmbH, Untersiemau, Germany) to spray coat the tablets. The geometry of a coated tablet is approximately 4 mm in height and 8 mm in diameter. A tablet was randomly selected after the following amounts of the sustained-release polymer were applied: 1.8, 3.6, 5.5, 7.3, 9.1, 10.9 and 12.7 mg/cm^2^ on a pilot-scale study.^17^ To facilitate for subsequent measurement comparisons, one side of each of the tablet was annotated by a scratch mark to serve as a datum.

### TPI Measurements

Terahertz pulsed imaging measurements on the top and bottom surfaces of the tablets were performed using a TPI Imaga 2000 system (TeraView Ltd., Cambridge, UK). At each measurement point, the terahertz radiation reflected from a tablet sample was recorded as a function of time over a scan range of a 2-mm optical delay. The TPI Imaga 2000 system is specifically developed for the fully automated scan of typical pharmaceutical solid dosage forms that usually have curved surfaces. A six-axes robot system was employed to handle the tablets. This ensures that the tablet is always at the terahertz focus position with its surface perpendicular to the terahertz probe during a TPI measurement.^18^ The terahertz radiation used here is broadband, covering a spectral range of 5–100 cm^−1^ (0.15–3 THz). The spot size of the focused terahertz beam at the tablet surface is estimated to be about 200 μm in diameter at its centre frequency of 1.5 THz (50 cm^−1^). For the accurate determination of the coating layer thickness, the refractive index of the coating matrix is required. The refractive index of the coating was measured by terahertz time domain spectroscopy using an uncoated tablet core as the reference. Using this method, a refractive index of 1.68–1.79 was determined^17^ and a value of 1.74 was used for thickness quantification.

### Spectral Domain Optical Coherence Tomographic Measurements

**Figure 1:**
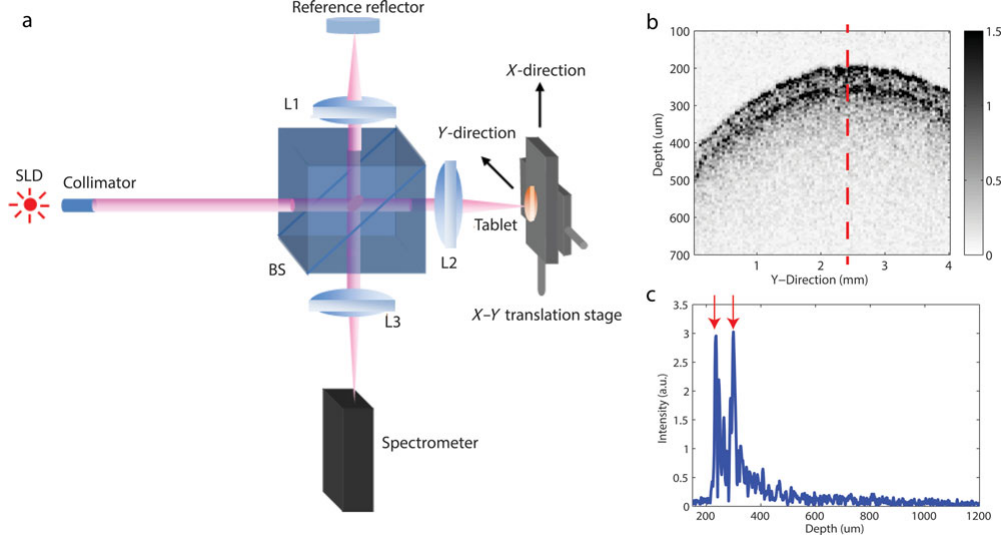
Schematic of an in-house SD-OCT system with a tablet placed on (a) X–Y translation stage. SLD, super-luminescent diode L1; L2, achromatic doublet; *f* = 30 mm; L3, achromatics doublet; *f* = 50 mm; BS, 50:50 beam splitter. The system acquired 100 × 100 B-scans, one of which is shown (b) with the tablet central region annotated by the dashed line and the respective A-scan with the interfaces annotated by the respective arrows shown (c).

The measurements were performed using an in-house SD-OCT system. As shown in Figure 1a; the light source used in the SD-OCT system was a low-coherence super-luminescent diode EXS210040 (EXALOS AG, Schlieren, Switzerland) centred at 840 nm and had a spectral full width at half maximum (FWHM) of 55 nm, giving rise to an axial resolution of 5.7 μm in air. The collimated light beam was split into two arms by a 50:50 beam splitter. Two identical achromatic doublets were placed in the reference and sample beam path to focus the light beam onto the reference reflector and the tablet surface, respectively. The back reflected light from the reference reflector and the tablet surface is recombined and allowed to interfere with each other at the exit of the beam splitter and is finally coupled into the Ocean Optics HR 2000+ spectrometer (Ocean Optics Inc., Dunedin, Florida) via another achromatic doublet for the subsequent detection.

In order to obtain multiple B-scans across the tablet surface, two translation stages arranged perpendicular to each other were used to move the tablet sample. In particular, raster scans were performed in a 4 × 4 mm^2^ area for the tablets’ top and bottom surfaces, respectively, to form an image map of the tablet. The mechanical scanning step size of the translation stage was 40 μm and a total of 10,000 pixels were acquired spatially for each of the respective surfaces. Despite the lower mechanical step size, the optical spot size on the tablet surface was measured to be 16 μm according to the FWHM. An example of B-scan acquired with the setup is shown in Figure 1b where an A-scan (Fig. 1c) from the central tablet region, annotated by the dashed line, is shown.

Film coating thickness evaluations were performed on selected B-scans at the central regions that are defined as the apex of the tablet curvature on the B-scan. The manual operation may simply involve computing the distance between pixel coordinates of the interfaces or alternatively, where accurate readings are required, peaks of the corresponding A-scans are manually matched with the interfaces on the B-scan where the film coating thickness may be deduced from the depth profile. The operation itself is understandably time-consuming and only a small area can be evaluated from each B-scan using this methodology. Thickness accuracy may also be limited because of the subjectivity of the definition of interface.

### Algorithm for Automated Data Analysis

**Figure 2:**
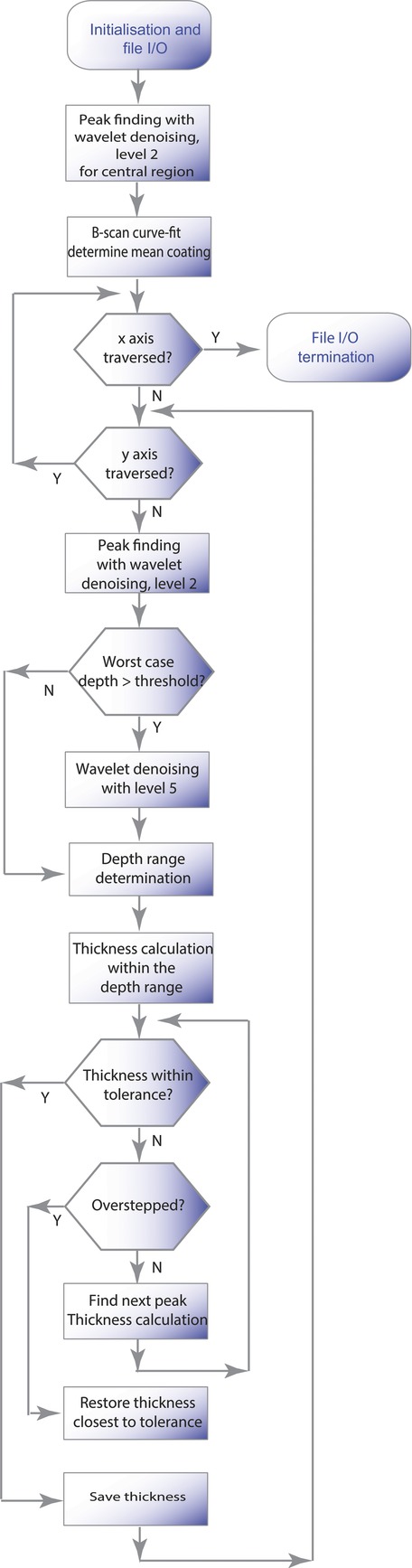
Data processing algorithm for automatically quantifying coatings on pharmaceutical tablets based on OCT measurements.

Film coating thickness of OCT measurements on pharmaceutical dosage forms, to date, has been quantified by means of visually identifying the layer interface in the OCT B-scans, obtained by post-processing OCT A-scans, also known as the depth profile. The layer thickness is then determined as the distance between the respective interfaces. However, because of scattering, the core/coating interface may be difficult to distinguish. This is one of the major challenges that is limiting applications of OCT in this area. Although this problem may be mitigated with the use of a light source operating at a longer wavelength (e.g., 1300 or 1550 nm), operating at a longer wavelength does not necessarily lead to a noticeable increase in the penetration depth.^10^ In contrast, the advantage of working with a shorter wavelength source gives rise to improved spatial and axial resolution,^10^,^12^ thus useful for studying coating uniformity. To alleviate film coating thickness uncertainties in the curved region of the tablet, where the sensor head is no longer at a normal angle to the surface, film coating thickness measurements are commonly only taken at the central region of the tablet. In the central region, the film coating thickness can be reliably measured as the optical beam is perpendicular to the tablet surface. The proposed algorithm as shown in Figure 2 seeks to automatically quantify the film coating thickness based on the A-scans.

#### Wavelet Denoising

Theoretically, an A-scan is composed of three terms: a DC component, an autocorrelation term arising from self-interference between different sample layers and a cross-correlation term because of interferences from the reference reflector and reflections from layer.^19^ It is the latter term of the A-scan that contains structural information of the sample. As the terms are a convolution with delta function, notable features in the sample therefore appear as peaks or local maxima on the respective A-scan. The strength of the peaks is proportional to the change in refractive index between the materials at the interface. Even though most polymers used in pharmaceutical coating fall into a relatively narrow band of refractive indices, as previous researches have shown,^10^–^16^ small differences in refractive index are typically sufficient to distinguish between different coating layers. Even though not used in the algorithm, a three steps moving average window filter may be used to pre-process the A-scans to accentuate the interface peaks while removing some of the scattering noise. However, it should be noted that by doing so, spatial resolution is compromised. The coating/core interface may still be difficult to distinguish because of scattering from the particles that make up the tablet powder compact or air inclusions and particles in the coating (≤100 μm). Owing to the much shorter wavelength (840 nm), the scattering noise in OCT measurements is stronger compared with terahertz imaging (300 μm at 1 THz). In order to separate the coating layer from the core as clearly as possible, the scattering noise needs to be reduced as much as possible prior to any further signals processing. Given that scattering originates from much smaller structures compared with the thickness of the coating layer, it will be possible to separate the scattering contribution in the frequency domain. By decomposing the A-scan into its respective frequency components, some high-frequency noise components can be removed, thereby isolating the remaining lower frequency peaks for thickness calculation. Instead of a simple Fourier transform, we use wavelet denoising to decompose the A-scans into a small number of wavelet coefficients that measure the correlation between the A-scan and a daughter wavelet 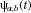, which is a scaled, by *a*, and shifted, by *b*, version of a mother wavelet 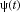. In particular, we used the undecimated wavelet transform to decompose the OCT A-scans into multilevel approximation 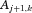 and detail coefficients 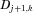, which are represented as follows:


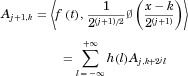
(1)


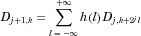
(2)

where 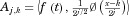, 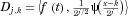, 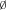 is the scaling function and *h* is the impulse response of low-pass paraunitary quadrature mirror filters.

**Figure 3:**
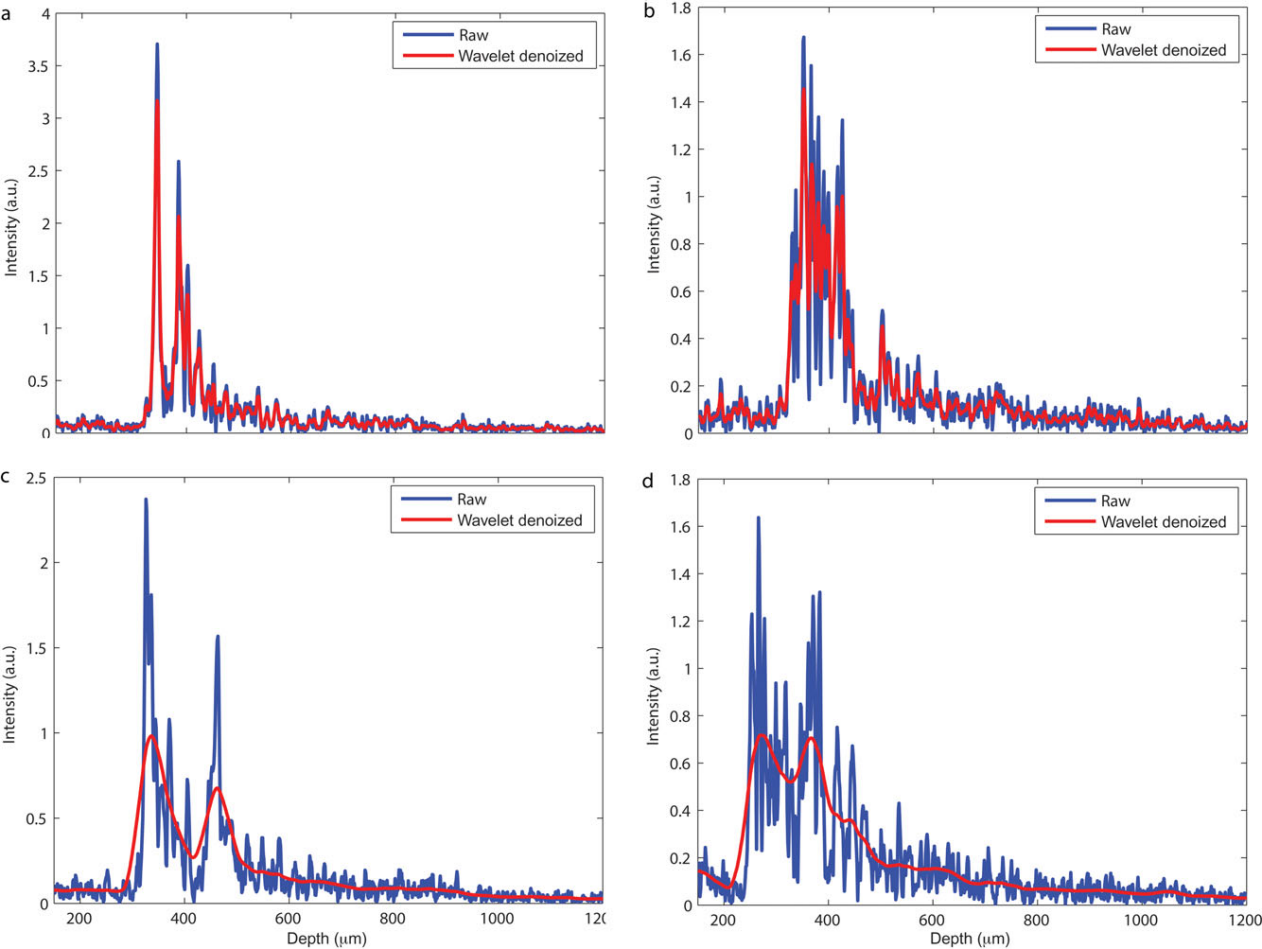
Examples of the raw A-scans and the wavelet denoised A-scans with decomposition levels of 2 and 5 for coating thicknesses 70 μm (a, b) and 140 μm (c, d), respectively.

The level of decomposition used is 2 and 5 for thin and thick coatings, respectively. The level of decomposition is obtained from performing a proof-of-concept analysis where the trade-offs between accurately identifying a suitable peak for thickness calculation and the peak convergence time is minimised. As an example, it was found experimentally that thick coatings performed better with an increased level of decomposition where the denoised A-scans show less high-frequency scattering noise, which facilitates subsequent peak finding. In contrast, such high decomposition level would not be applicable for thin coatings that would effectively filter out the peaks arising from the coating core interface. To demonstrate the effect of denoising so as to isolate the peaks originating from the interfaces from the particle scattering, Figure 3 compares two examples of raw and wavelet denoised A-scans for a tablet with 70 and 140 μm coating thickness, respectively. Specifically, Figures 3a and 3b show the comparison for the 70-μm tablet, whereas Figures 3c and 3d shows the comparison for the 140-μm tablet. In general, although alternative filtering methods may lead to similar improvements, wavelets are practically advantageous because no prior knowledge of the interfering non-stationary noise is required. Furthermore, wavelets are also superior in terms of functions reconstruction than Fourier transform processing routines that are used in conventional filter design because of the inherent localised nature of wavelet basis function in both time (or space) and frequency (or spatial frequency). This is in contrast with Fourier transform, which uses infinite sinusoids as basis functions.

#### Interface Detection

**Figure 4:**
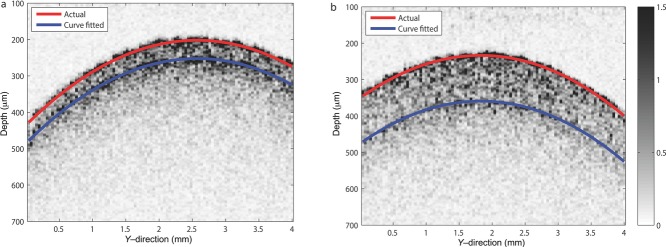
B-scans for tablets with coating thicknesses of 70 μm (a) and 140 μm (b), respectively, where the air–coating and coating–core interfaces are curve-fitted using the peaks identified from the wavelet denoised A-scans.

From the wavelet denoised A-scan, the peaks arising from the air–coating interface and coating–core interface can be established more readily. Exploiting the fact that wavelet coefficients from real features tend cluster spatially as opposed to speckle noise,^20^ the interface may be identified by curve-fitting the peaks from the respective A-scans on the B-scan with the least absolute residuals method. Closer inspection of the obtained B-scans reveals that the shape of the coating core interface generally resembles the air–coating interface, the coating–core interface on the B-scan can be expressly obtained by fitting the corresponding denoised peaks using the air–coating fit, where the intercept fitting variable denotes the distance between interfaces or effectively the mean coating thickness. Alternatively, the interfaces may be obtained by fitting a circle that has been demonstrated to improve the robustness.^16^ Figure 4 shows the outline of the air–coating and coating–core interfaces, respectively, on the B-scan. The root mean square error (RMSE) for curve-fitted air–coating interface for the 70 and 140 μm coating thickness is 4.1 and 4.3 μm, respectively, whereas the RMSE for the coating–core interface is 2 and 6.8 μm. The extracted mean coating thicknesses from the fits are 62 and 157 μm, respectively. It should be noted that the presented B-scans have a spatial resolution of 40 μm constrained by the stepping size of the motorised mechanical delay as opposed to the theoretical resolution limited by the optical spot size of 16 μm. In general, the stepping size of motorised mechanical delay can go down to as small as 1 μm and therefore is not a hindrance for measurement. The advantage of having a larger step size, however, is the significant reduction of the measurement time by at least an order of magnitude in this instance.

#### Peak Finding

**Figure 5:**
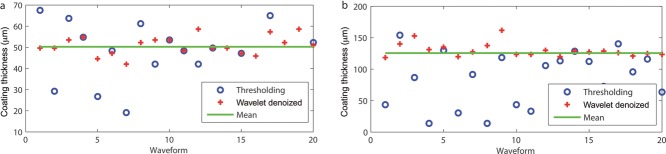
Examples of coating thickness measurements extracted with the standard peak finding approach by means of simple thresholding and our proposed algorithm for A-scans at several example points on the OCT map for coating thicknesses 70 μm (a) and 140 μm (b), respectively.

Even though the wavelet denoising can remove a lot of the scattering noise, the resulting signal is typically still too noisy at the coating–core interface for clear thickness quantification. In the case of manual thickness evaluation, the A-scans are visually compared with neighbouring A-scans from the same B-scan that has been randomly selected from the central regions where peaks are identified on the basis that would give rise to a similar coating thickness value. To replicate such an operation into the algorithm, an iterative peak finding method is used to process the A-scans in order to converge at the peaks that would lead to a thickness value within a tolerance value to the mean coating thickness. Specifically, the peak finding method looks for local maxima where candidate peaks are differentiated from neighbouring peaks based on magnitudes within a prescribed depth range obtained from the mean thickness. As an example, where a peak has been identified because of high magnitude, if the resultant coating thickness value exceeds the coating thickness tolerance (such as ±5 μm), the candidate peak would be rejected and the neighbouring peaks would be inspected to converge at a peak that would result with a thickness within the thickness tolerance. However, when the leap between neighbouring peaks becomes too large leading to diverging coating thickness, the algorithm backtracks to a previously identified peak with a thickness value that is closest to the thickness tolerance. Figure 5 compares the converged coating thicknesses as determined using the proposed peak finding algorithm against the coating thickness determined using simple magnitude thresholding. In order to identify the peak for thin coatings, the algorithm iteratively traverses the local maxima to identify the relevant peaks that would produce minimum error when compared with the mean thickness of the central region obtained from curve-fitting shown as the green line in Figure 5. By comparing the converged coating thickness from the proposed algorithm (red crosses) against the coating thickness derived using the simplistic thresholding method (blue open circles), it is evident that the peaks with thickness values closer to the median value have been identified. The algorithm is developed under the Matlab environment with the Wavelet Toolbox (Matlab R2012; The MathWorks Inc., Natick, Massachusetts).

### Thickness Validation

The coating thickness values computed using the algorithm are validated against the coating thickness measurements determined manually from the B-scans as well as by TPI. Specifically, coating thickness via manual visual inspection is performed on a randomly selected B-scan with the air–coating interface and coating–core interface is visually identified to determine the distance between the interfaces at the central region.

As it is difficult to orientate the captured OCT B-scan with 40 μm spatial resolution to the TPI image of a spatial resolution (200 μm) as would be required for pixel by pixel comparison, the aggregate coating thickness measurements from the respective methods are compared. Even though it is possible to determine the refractive index of the coating material at terahertz frequencies for thickness computation, the refractive index at optical frequencies remains unknown throughout in this investigation. Such information may, however, be obtained via suitable spectroscopic methods such as spectroscopic ellipsometry.^21^ Without knowledge of the refractive index at optical frequencies, the absolute coating thickness cannot be determined from the OCT measurements. However, as the refractive index of the coating material does not generally change with the amount of coating material applied, validation can be achieved by observing a constant factor between the OCT and TPI coating thickness measurements over tablets with a range of coating thickness when the same refractive index is used for OCT and TPI thickness calculation. The constant factor would therefore correspond to the ratio between the coating refractive index at terahertz and optical frequencies, respectively.

## RESULTS AND DISCUSSIONS

### Thickness Validation

**Figure 6:**
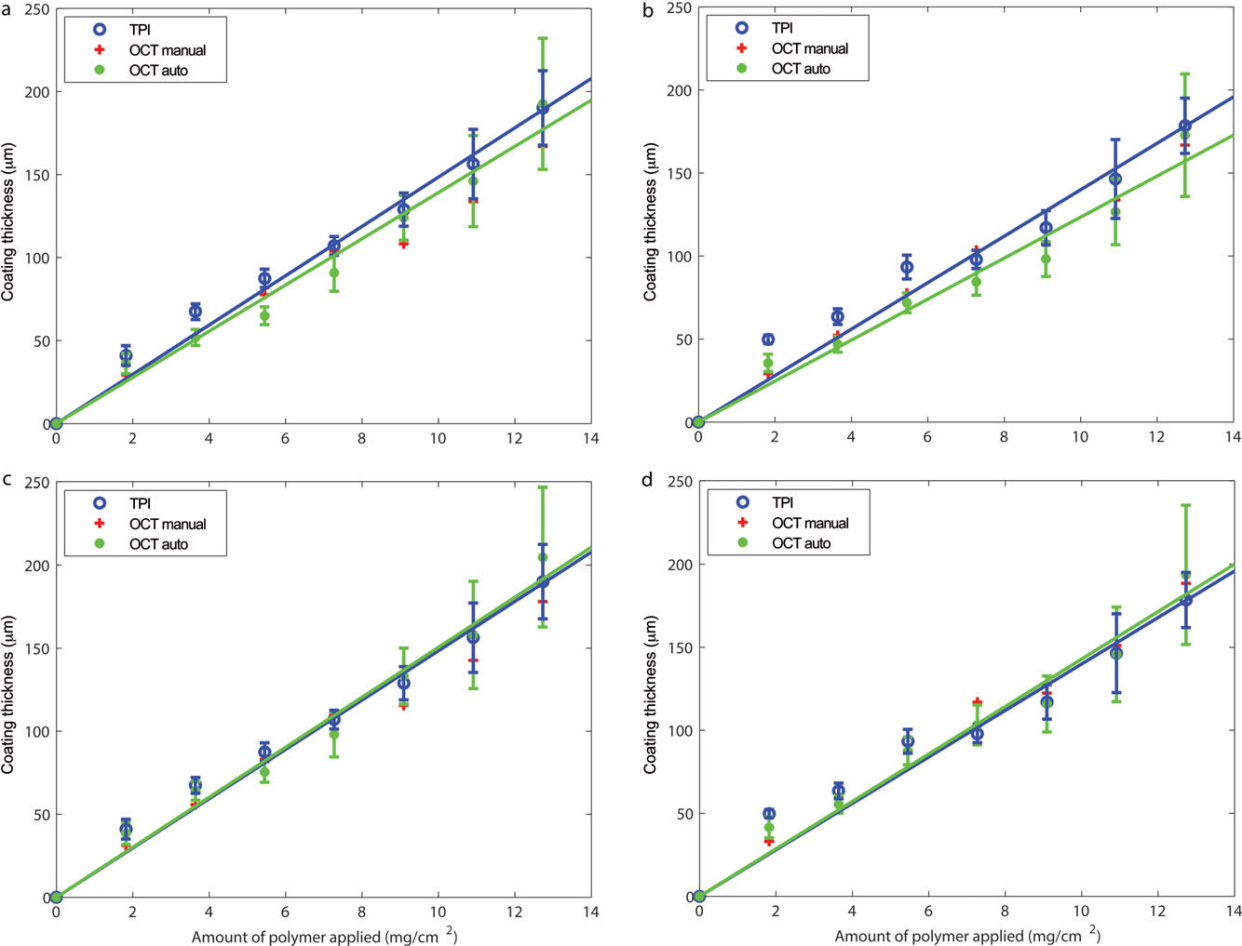
Comparison of the pharmaceutical tablet coating thicknesses measured using TPI and OCT, where the OCT measurements are quantified by manual visual inspection and the proposed automated algorithm using the same refractive index as TPI in (a) and (b) and with the estimated refractive index (c) and (d) for the top and bottom surface of the bi-convex tablet, respectively. Lines are plotted to guide the eye.

Figures 6a and 6b compare the TPI measured tablet coating thickness with the coating thickness obtained by manual analysis as well as the automatic algorithm for both the tablets’ top and bottom surfaces, respectively. For coating amounts less than 5.5 mg/cm^2^, there is poor overlap between coating thickness determined by the automatic algorithm and the TPI measurement. This can be attributed to the value of the refractive index used in the OCT thickness calculation. To estimate the refractive index of the coating material at the optical frequencies, both the TPI and the OCT thickness values are linearly fitted with least squares to obtain values of the slope. The fitting achieved a RMSE for the OCT measurements of 8.3 and 10 μm for top and bottom surface, respectively, whereas the RMSE for the TPI measurements was 9.4 and 13 μm. The ratio between the slopes, which in this case is 1.06 and 1.13 for the top and bottom surfaces, respectively, is indicative of the ratio between the refractive index of the coating material at terahertz and optical frequencies. In general, small variations in refractive index between top and bottom surfaces on dosage forms are not uncommon as encountered in previous investigation.^9^ Figures 6c and 6d show the corrected OCT thickness based on the estimated refractive index at optical frequencies and there is a good agreement between the thickness values obtained from using different methods. In particular, there is a good match between manual and automatic OCT measurements despite discrepancies between how the measurements are processed. In particular, owing to the laborious nature of manual analysis, coating thickness measurements are taken from only the central regions of a single B-scan that has been pre-processed by means of a moving average filter. In contrast, the algorithm operated on the raw A-scans from all B-scans. Also evident from Figure 6 is that the coating thickness standard deviation appears to be larger for thicker coatings, such as for greater than 9.1 mg/cm^2^. This is primarily because of the fact that the strength of the reflected signal from the coating–core interface decreases with increasing coating thickness. For very thick coatings, the strength of the reflected signal from the coating–core interface may be smaller in magnitude than the scattered signals from inside the tablet matrix. This therefore hinders the algorithm from accurately discriminating the reflection signal originating from the interface from the scattered signals and hence leads to relatively larger standard deviation of the extracted coating thickness. TPI in contrast, operates at a longer wavelength and is less susceptible to scattering and hence is able to measure thick coatings reliably, whereas OCT is more suitable for precisely charactering thin coatings. It should also be highlighted that the degree of sample surface roughness would also contribute to variations in refractive index measurement because of signal attenuations from scattering losses.

### Measurement Uncertainty

Optical coherence tomography measurements were performed perpendicular to the overall tablet surface plane at the tablet centre as opposed to being perpendicular to the tablets’ curved surface as in the case of the TPI measurement using a six-axis robotic arm. Consequently, the OCT measured coating thickness is systematically larger than the actual coating thickness as they do not account for the surface curvature of the tablet. The measurement error arising from not measuring perpendicular to the tablet surface has previously been studied.^9^ For a round bi-convex tablet, geometrical reconstruction of the tablet is possible by forming two spherical caps from a circle and a cylinder without top or bottom.^22^ With a calculated radius of curvature of 8.5 mm and a worst-case scenario measurement distance from the central region of 3 mm, the worst-case error is approximately 6.4%.

As the optical beam was focused onto the central region of the tablet, it is understandable that as the beam scans across an increasingly curved surface of the tablet, there is the uncertainty as to whether the depth of field of the focusing lens would be sufficient for the depth profiles across the tablet. The lens used in the measurement has a focal length of 30 mm and a depth of field of 800 μm. As the measurement in this study spanned across an area of 4 mm by 4 mm laterally, the additional vertical distance, relative to the surface plane of the tablet central region, introduced as a result of tablet curvature would be 182 μm for a worst-case 3-mm lateral measurement distance from the central region. As the thickest tablet coating layer is approximately 200 μm in this study, the vertical distance in the worst-case scenario would be 382 μm in total that is shorter than the theoretical depth of field 800 μm of the lens used in the present investigation. Therefore, the depth of field of the focussing lens is sufficient for the coating thickness range investigated in this study.

### Intra-Tablet Coating Uniformity

**Figure 7:**
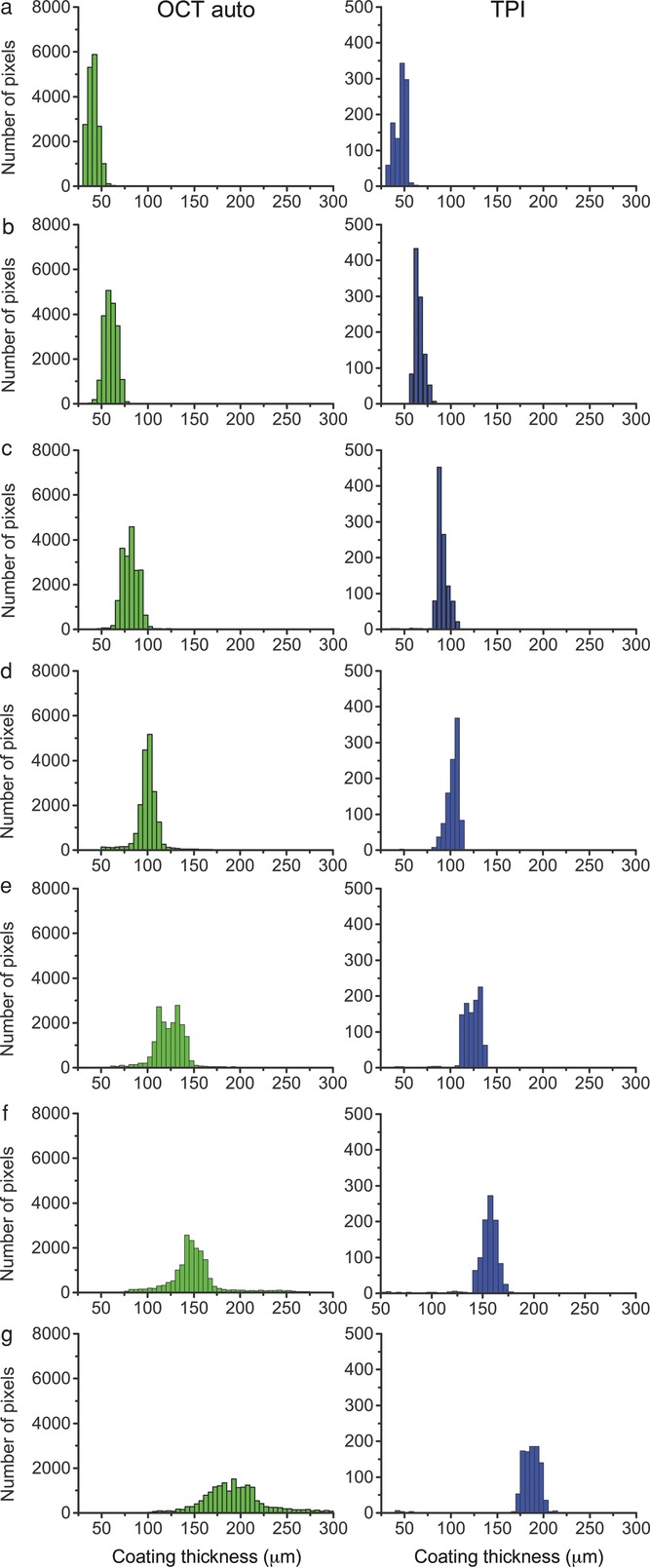
Comparison of the intra-tablet coating thickness distribution measured using TPI and OCT for one of the surfaces of the bi-convex tablet.

Optical coherence tomography has some advantages over TPI in terms of the achievable spatial resolution and data acquisition rate, which is at least an order of magnitude higher compared with TPI leading to more data points to assess coating uniformity. Figure 7 compares the intra-tablet coating thickness distribution for the different coating thicknesses that were measured using OCT and TPI. Because of the higher data acquisition rate and a smaller optical spot size as described above, the histograms of the OCT coating thickness measurements is constructed from more data points (10,000 pixels), a 25-fold increase compared with the TPI measurements (400 pixels). As expected, the histograms also follow the same normal distribution but with a greater variance. The smaller variance of the TPI measured coating thickness histograms is attributed to a comparatively larger terahertz spot size, 200 μm, as opposed to an optical spot size of 16 μm, leading to spatial averaging over at least two order of magnitudes more surface area. The possibility to sample tablet surfaces at finer resolutions is an advantage of OCT compared with TPI.

### Measurement Reliability

**Figure 8:**
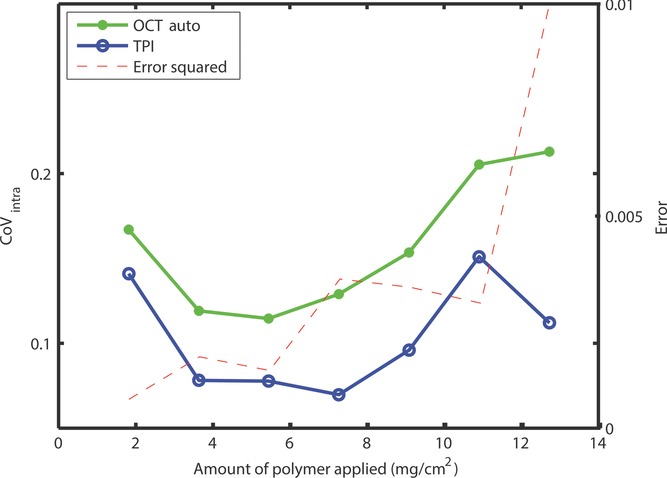
Comparison of the intra-tablet variability as measured using TPI and OCT for the bi-convex tablet.

It is important to assess the accuracy of the OCT measurements, in particular in terms of the increasing scattering losses with increasing coating thickness. Specifically, the intra-tablet coating variability^23^ (CoV) that is defined as the ratio of the standard deviation of the film coating thickness to the mean film thickness over the tablet's surface (CoV = *σ*/*μ*) is computed for both OCT and TPI measurements for the tablets shown in Figure 8. As can be seen, coating variability obtained by OCT measurements closely follows the coating variability benchmarked by TPI for coating amounts less than 5.5 mg/cm^2^ for both surfaces. Above this coating amount, there appears to be less consistent correlation between the variability measured with the respective techniques. To further investigate this, we plotted out the squared error between the variability (CoV_OCT_ − CoV_TPI_)^2^ where we can observe that the error is smallest and remains approximately constant for coating less than 5.5 mg/cm^2^. It is interesting to note a general monotonic rise in error for coating thickness thereafter. Although we can generally expect a higher variability from the OCT measurements as opposed to TPI because of a smaller spot size as described above, it would not constitute as the main cause behind the monotonic rise in error. As the error between the variability measured using OCT and TPI increases monotonically for thicker coatings (>100 μm), OCT is most likely to be used for analysis of thin coatings. We understand that the thickness limit would realistically depend on the measurement setup and the matrix of pharmaceutical dosage form, this finding appears to be consistent with existing OCT studies on pharmaceutical dosage forms where the reported coating thickness measurements are all less than 100 μm.^10^–^13^ For coating thickness greater than this identified limit, TPI is recommended and therefore could complement OCT for investigations on thick pharmaceutical coatings as also suggested in the previous study.^11^

## CONCLUSIONS

With the emergence of OCT as the suitable modality for the non-destructive evaluation of pharmaceutical coatings, we have evaluated the SD-OCT against the more established TPI for the purposes of film coating quantification. To facilitate a comprehensive evaluation, we proposed and validated an algorithm to automatically quantify coatings of a range of thickness from the acquired OCT A-scans. Although we also demonstrated the capability of OCT to obtain more information on intra-tablet coating thickness uniformity than previously possible with TPI, our findings show that the additional intra-tablet variability information would only be useful for coating thickness less than 100 μm, above which measurement reliability becomes a concern.
